# A System of Operative Surgery

**Published:** 1910-06

**Authors:** 


**A System of Operative Surgery.** Edited by F. F. BURGHARD, M.S., F.R.C.S. Vol. III. Pp. xxxii, 764. London: Oxford Medical Publications. 1909.

This volume is one of the best of the series. The subject of the removal of tuberculous glands is by Mr. Harold Stiles, and it is notable for the very exact anatomical description and the beautiful illustrations of the same. The incisions necessary in the neck are carefully planned so as to avoid the unsightly scars so often seen in front of the sterno-mastoid. The surgery of the spleen is by Messrs. Moynihan and Upcott, and that on the thyroid gland by Mr. Berry. The latter is perhaps somewhat marred by the inclusion of such procedures as injection of thyroid tumours and exothyropexy at some length, which the author would never employ himself. The statement that chloroform is to be the anæsthetic of choice in extirpation of thyroid tumours is one which will provoke much criticism. The questions, too, of surgical treatment of exophthalmic goitre, the rôle of the parathyroids and thyroid grafting, are not mentioned.

Mr. Mayo Robson deals with the surgery of the bile passages and the pancreas, and it is rather remarkable that liver surgery, apart from that of the bile-ducts, is omitted. The conditions which require or justify removal of the gall-bladder are considered to be very limited. Both these chapters form a very full and yet concise account of the subjects of which they treat.

The chapter on brain surgery is by Mr. Rawling, and it is consistently good. The hand trephine and Gigli's saw are considered to be the most useful instruments in opening the skull. Mr. Thorburn writes on spinal surgery, and his account of the whole subject is the best with which we are acquainted. Dr. David Newman writes on the operations on the kidneys and ureters, and, whilst the note of personal opinion from such an authority is of great importance, it involves an unfortunate omission of much that represents the current teaching of the day. This is notably the case in the instance of methods of determining the functional efficiency of both kidneys, which are not mentioned. Suppurative lesions of the kidney are treated on very conservative principles, nephrotomy being advised as the method of choice in such cases.

Mr. Thompson Walker deals with operations upon the bladder, and all the details given, both of operative technique and after-treatment, are very valuable.

Mr. Freyer's articles on vesical calculus and the surgery of the prostate call for no special comment. The latter is characterised by the same undue optimism that the author has always displayed in writing on this subject. To say that "the success attending suprapubic enucleation means an absolute cure, the patient regaining the power of retaining and passing urine naturally without the aid of a catheter as well as ever he did," is not in accordance with the facts as ordinarily seen by operating surgeons.

Mr. Walker's article on the urethra is especially valuable because of the large number of statistics and references to operative results. The favourable mention of the excision of strictures is to be noted, as it is to be desired that this method should be much more widely practised than it is. Mr. Burghard's article on the male generative organs contains a good account of the operations for hypospadias, but the meagre account of the operations for malignant disease of the testis, without any reference to the removal of the lymphatics, is a matter for regret. The article on operations for cancer of the breast, by Mr. Stiles, is quite up to his usual standard, but other operative procedures on the mamma are very briefly noticed.

The volume concludes with an article by Mr. Godlee upon thoracic operations. The methods of positive and negative pressure are discussed and illustrated, and the newer operations, *e.g.* cardiolysis and the removal of pulmonary emboli, described. This section would be still more valuable if it were not so brief.

